# Reply to Katkuri et al

**DOI:** 10.1093/icvts/ivag015

**Published:** 2026-02-04

**Authors:** Robert Pruna-Guillen, Thanakorn Rojanathagoon, Aung Oo, Ana Lopez-Marco

**Affiliations:** Department of Cardiothoracic Surgery, St Bartholomew’s Hospital, London EC1A 7BE, United Kingdom; Department of Cardiothoracic Surgery, St Bartholomew’s Hospital, London EC1A 7BE, United Kingdom; Department of Cardiothoracic Surgery, St Bartholomew’s Hospital, London EC1A 7BE, United Kingdom; William Harvey Research Institute, Queen Mary University, London, United Kingdom; Department of Cardiothoracic Surgery, St Bartholomew’s Hospital, London EC1A 7BE, United Kingdom; William Harvey Research Institute, Queen Mary University, London, United Kingdom

We thank Sharma et al^1^ for their reading of our article^2^ and for their constructive comments on study design and reporting. We welcome the opportunity to respond and to further contextualize our findings.

First, we agree that our evaluation is an observational, single-centre, uncontrolled before–after comparison undertaken within a departmental service redesign. As stated in the Methods, Discussion, and Limitations, implementation of the On-Call Specialist Aortic Rota occurred alongside updates in operative strategy, perfusion management, and perioperative pathways. Accordingly, we framed our results as associative rather than causal, acknowledging risks of confounding, learning-curve effects, secular trends, and calendar-time bias. We, therefore, concluded that these findings require confirmation in multicentre cohorts using robust quasi-experimental designs. Nonetheless, reporting outcomes after service reconfiguration remains valuable in acute type A aortic dissection, where randomized evaluation of organizational change is rarely feasible.

Second, regarding covariate adjustment, we chose a multivariable mortality model a priori to respect the limited number of outcome events and to minimize overfitting. Over-parameterization in logistic regression is known to produce unstable coefficients, exaggerated effect sizes, and inflated type I error, particularly when events are limited relative to the number of predictors.^3^ Age and previous cardiac surgery were chosen a priori based on established prognostic importance and supported by univariable screening, as detailed in the Statistical Analysis section. The inverse probability of treatment weighting (IPTW) analysis was performed as a complementary approach to address confounding from non-random group assignment, incorporating a broader set of pretreatment variables and achieving acceptable covariate balance (standardized mean differences <0.1). The resulting average treatment effect estimate was directionally concordant with the multivariable regression, providing triangulation across analytic approaches. The use of multiple complementary adjustment strategies is explicitly recommended in observational effectiveness research to assess the robustness of findings to modelling assumptions. While no analytic method can fully eliminate residual confounding in non-randomized designs, concordant results across regression-based and weighting-based approaches strengthen confidence that the observed association is not solely an artifact of model specification.^4^^,^^5^ We agree that explicit discussion of estimands and causal structure can further enhance transparency, and this is an important methodological consideration for future evaluations of organizational interventions.

Third, we appreciate the opportunity to clarify reporting points. Preoperative creatinine was non-normally distributed; therefore, between-group comparisons used a non-parametric test, explaining the numerical difference alongside a non-significant *P*-value. Tracheostomy is presented as a crude rate in Table 2, whereas adjusted associations are derived from regression; clearer separation of crude versus adjusted estimates would indeed reduce confusion. Finally, the cardiopulmonary bypass and cross-clamp coefficients in Table 3 represent mean differences in min (linear models), not odds ratios, and we regret any ambiguity. Finally, our data reinforce that early outcomes in ATAAD are predominantly driven by preoperative acuity. In our multivariable Cox analysis, cardiogenic shock or tamponade was independently associated with mortality, underscoring that disease severity, rather than operative duration or extent of repair, remains a principal determinant of early risk.

We are grateful to Sharma et al. for their critique, which highlights both the challenges and the importance of evaluating service redesign in emergency aortic surgery. We hope these clarifications strengthen interpretation of our work and support future efforts to build even higher-quality robust evidence on specialist aortic rotas.
